# Synthesis and Insecticidal Activity of an Oxabicyclolactone and Novel Pyrethroids

**DOI:** 10.3390/molecules171213989

**Published:** 2012-11-26

**Authors:** Elson S. de Alvarenga, Vânia M. T. Carneiro, Gabriela C. Resende, Marcelo C. Picanço, Elizeu de Sá Farias, Mayara Cristina Lopes

**Affiliations:** 1 Department of Chemistry, Federal University of Vicosa, Av. P. H. Rolfs, s/n, 36570-000, Vicosa, MG, Brazil; 2 Department of Animal Biology Animal, Federal University of Vicosa, Av. P. H. Rolfs, s/n, 36570-000, Vicosa, MG, Brazil

**Keywords:** oxabicyclo, photochemistry, furfural, insecticide, pyrethroid

## Abstract

Deltamethrin, a member of the pyrethroids, one of the safest classes of pesticides, is among some of the most popular and widely used insecticides in the World. Our objective was to synthesize an oxabicyclolactone **6** and five novel pyrethroids **8**–**12** from readily available furfural and D-mannitol, respectively, and evaluate their biological activity against four insect species of economic importance namely *A. obtectus*, *S. zeamais*, *A. monuste orseis*, and *P. americana*. A concise and novel synthesis of 6,6-dimethyl-3-oxabicyclo[3.1.0]hexan-2-one (**6**) from furfural is described. Photochemical addition of isopropyl alcohol to furan-2(5*H*)-one afforded 4-(1'-hydroxy-1'-methylethyl)tetrahydro-furan-2-one (**3**). The alcohol **3** was directly converted into 4-(1'-bromo-1'-methylethyl)-tetrahydrofuran-2-one (**5**) in 50% yield by reaction with PBr_3_ and SiO_2_. The final step was performed by cyclization of **5** with potassium *tert-*butoxide in 40% yield. The novel pyrethroids **8**–**12** were prepared from methyl (1*S*,3*S*)-3-formyl-2,2-dimethylcyclopropane-1-carboxylate (**7a**) by reaction with five different aromatic phosphorous ylides. Compounds **6**–**12** presented high insecticidal activity, with **6 **and **11** being the most active. Compound **6** killed 90% of *S. zeamais* and 100% of all the other insects evaluated. Compound **11** killed 100% of all insects tested.

## 1. Introduction

**Figure 1:**
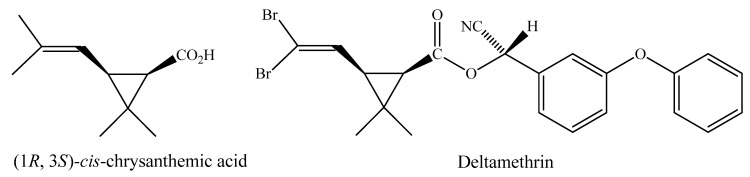
Structure of the (1*R*, 3*S*)-*cis*-chrysanthemic acid.

The term pyrethroid is used to designate synthetic insecticides derived structurally from the pyrethrins. Decades of research done by the agrochemical industry, government, and academic laboratories have resulted in a wide range of new pyrethroids and in a multiplicity of uses in agriculture, veterinary medicine and control of domestic pests. Few classes of biologically active compounds have such great potential for structural variation with retention or enhancement of potency [1,2]. Deltamethrin, a member of one of the safest classes of pesticides, is among some of the most popular and widely used insecticides in the World. This pyrethroid, which is derived from (1*R*, 3*S*)-*cis*-chrysanthemic acid (Figure 1), was first prepared by Elliott and Janes [3]. 

Pyrethroid insecticides have been used for more than 40 years and are sold as mixtures containing a combination of two or more compounds. Most of the pyrethroids registered for use in the World in a large variety of agricultural products are derived from *cis*-chrysanthemic acid. The toxicity of DDT urged the development of pyrethroids which rapidly knock down flying insects and have negligible persistence in the environment. Moreover, an important feature of the pyrethroids is that they present low toxicity to mammals and birds [4,5,6,7,8,9].

Because of the utility of the oxabicyclo moiety as a building block for the construction of *cis*-chrysanthemic acid as well as other natural products and biologically active compounds, there has been much interest in the synthesis of 6,6-dimethyl-3-oxabicyclo[3.1.0]hexan-2-one [10,11,12,13,14,15,16,17,18,19,20,21].

The insecticidal activity of eight pyrethroids were evaluated against four insect species of economic importance namely *Acanthoscelides obtectus *(Say) (Coleoptera: Bruchidae), *Sitophilus zeamais *Mots. (Coleoptera: Curculionidae), *Ascia monuste orseis* Latr. (Lepidoptera: Pyralidae), and *Periplaneta americana* (L.) (Dictyoptera: Blattidae). *A. obtectus* and *S. zeamais* are cosmopolitan grain store pests mainly found in bean and maize, respectively. *S. zeamais* has been causing great damages in several fruit cultures, such as apple, peach trees, and grapevines in the South of Brazil [22,23]. The kale leaf worm, *A. monuste orseis*, is a key pest of Brassicaceae (kale, cabbage, cauliflower, broccolis, mustard and radish), mainly in subtropical and tropical regions [24]. The cockroach, *P. americana*, is a domestic pest in tropical countries. Cockroaches are urban pests of major importance because of their appearance in household products and the transmission of diseases. They are a common pest of restaurants, bakeries, grocery stores, *etc.* In this paper we describe a novel synthetic route to the 6,6-dimethyl-3-oxabicyclo[3.1.0]hexan-2-one from the readily available furfural in four steps. Five novel pyrethroids have been prepared also from D-mannitol and biological assays of the synthesized compounds were conducted with *A. obtectus*, adults of *S. zeamais*, second-instar larvae of *A. monuste orseis*, and second-instar nymph of *P. americana*.

## 2. Results and Discussion

### 2.1. Synthesis

**Scheme 1:**
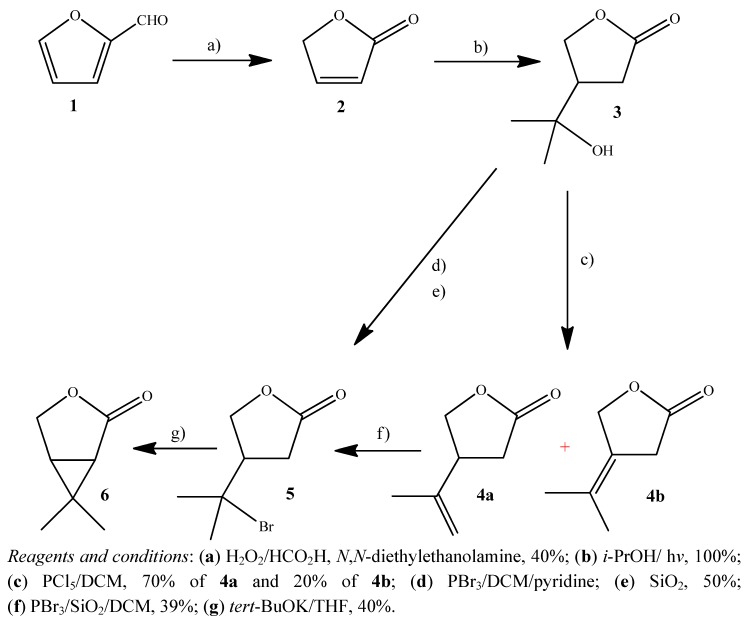
Synthesis of 6,6-dimethyl-3-oxabicyclo[3.1.0]hexan-2-one (**6**).

The butenolide furan-2(*5H*)-one (**2**) was obtained by performic acid oxidation of furfural (**1**) in the presence of *N*,*N*-diethylethanolamine [25]. Irradiation of furan-2(5*H*)-one in isopropyl alcohol with four low pressure mercury lamps of 15 W each (LPML) afforded the adduct **3** in quantitative yield (Scheme 1). The excited butenolide **2** abstracts a hydrogen from the isopropyl alcohol and the alcohol radical adds to the double bond β to the carbonyl of the butenolide (1,4-addition) to afford the alcohol **3** [26].

Dehydration of the alcohol **3** with PCl_5_ in anhydrous DCM was carried out in 90% yield. The isomeric alkenes **4a** and **4b** were not fully separated from each other by flash column chromatography. Only a small fraction of each isomer was obtained for the spectrometric identification and insecticidal evaluation. The mixture of the alkenes containing the major isomer **4a** (as estimated by ^1^H-NMR spectroscopy) was used in the next step of the synthesis.

Addition of HBr to the double bond of alkenes **4a** and **4b** afforded only 4-(1'-bromo-1'-methylethyl)tetrahydrofuran-2-one (**5**) in 39% yield. The reaction was carried out with PBr_3_/SiO_2_ in DCM, which was adapted from the methodology described for other compounds by Sanseverino and Mattos [27]. This methodology has the advantage of avoiding the need of drying chemicals, rigorous exclusion of moisture, light, and oxygen from the reaction media, and generation of dry HBr.

Trying to optimize the synthesis we envisaged a direct conversion of the alcohol **3** into the bromide withough having to prepare the alkenes, therefore avoiding one step. The alcohol **3** was thus treated with PBr_3_ and SiO_2_ in DCM to afford 4-(1'-bromo-1'-methylethyl)tetrahydrofuran-2-one (**5a**) in 50% yield. In addition to saving one step, the yield was increased by 15% in comparison with the dehydration followed by addition of HBr (35% yield for the two steps).

Treatment of compound **5** with potassium *tert*-butoxide in anhydrous THF gave 6,6-dimethyl-3-oxabicyclo[3.1.0]hexan-2-one (**6**), in 40% yield. The strong base removed the α-proton to the carbonyl group to form a stabilized carbanion. The positively charged carbon (formed after displacement of the bromine) is attacked by the negative carbon (stabilized carbanion) to afford the oxabicyclo compound **6**. The mechanism of nucleophilic substitution unimolecular is favored in this reaction by formation of the relatively stable tertiary carbocation.

The carbonyl from the lactone is characterized by the intense band in 1774 cm^−1^ in the infra-red and by the signal in 175.3 ppm in the ^13^C-NMR spectrum. The complete assignment of the signals in the NMR spectra was carried out with the aid of COSY and HETCOR. All the other data from infra-red, mass and NMR spectroscopies add up confirming the structure of the oxabicyclo.

**Scheme 2:**
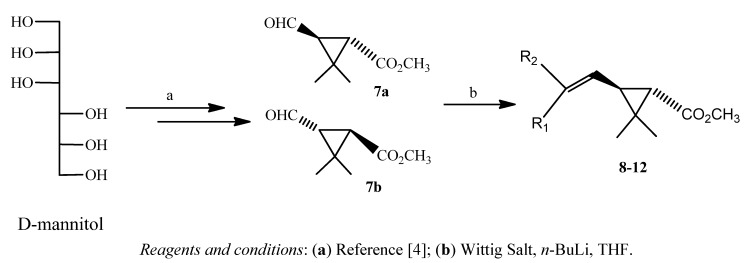
Synthesis of pyrethroids **8**–**12** from D-mannitol.

The preparation of novel pyrethroid methyl esters from the readily available D-mannitol is depicted in Scheme 2. The convertion of D-mannitol to aldehyde (**7**) is described in the literature [4].

Five different phosphorus ylides were employed to prepare ten novel pyrethroids **8**–**12** from aldehyde **7** in yields which varied from 45 to 65% (Table 1). 

**Table 1:** Synthesis of the pyrethroids **8**–**12** from **7**: reaction conditions and yields.

Pyrethroids	Wittig salt	R_1_	R_2_	Proportion	Yield (%)
				*Z* : *E*	
**8a**	*o*-MeOPhCH_2_PPh_3_^+^Cl^−^	-H	*o*-CH_3_OPh	1 : 1	46
**8b**		*o*-CH_3_OPh	-H		
**9a**	*m*-MeOPhCH_2_PPh_3_^+^Cl^−^	-H	*m*-CH_3_OPh	1 : 1	45
**9b**		*m*-CH_3_OPh	-H		
**10a**	*o*-ClPhCH_2_PPh_3_^+^Br^−^	-H	*o*-ClPh	2 : 1	64
**10b**		*o*-ClPh	-H		
**11a**	F_5_PhCH_2_PPh_3_^+^Br^−^	-H	F_5_Ph	1 : 1	50
**11b**		F_5_Ph	-H		
**12a**	* *p*-EtOPhCH_2_PPh_3_^+^Cl^−^	-H	*p*-(CH_3_CH_2_O)Ph	1 : 1	65
**12b**		*p*-(CH_3_CH_2_O)Ph	-H		

* (4-Ethoxybenzyl)triphenylphosphonium chloride **9g** was acquired from Sigma-Aldrich.

The close retention factors of the diastereomeric pyrethroids hampered their separation by flash column chromatography. The alkenes were characterized by FTIR, NMR, and mass spectrometry as a mixture of isomers. Microanalytical data of oils are usually unsatisfactory thus the assessments of the purity of the oily pyrethroids were carried out by TLC and high field NMR.

### 2.2. Bioassay

Table 2 presents the biological assays of compounds **6**–**12**, permethrin and the control sample (acetone) for *A. obtectus*, *S. zeamais*, *A. monuste orseis*, and *P. americana. *All evaluated substances showed insecticidal activity against the four species of pests since they caused significant mortality (*p* < 0.05), higher than the control (acetone). When the substances are compared with the standard of efficiency (permethrin) we found that all substances caused high mortality to *A. obtectus* (mean 97.5% 24 h after application).

Substances **6** and **8**–**12** presented high mortality to *S. zeamais* (mean 95.42% 24 h after application) and larvae of *A. monuste* (mean 97.08% 24 h after application). Moderate activity for substances **7a **(45.00% mortality) and **7b **(42.50% mortality) and high activity for all the other substances were observed after 12 h of treatment against *S. zeamais*. Mortality reached 100% after 24 h for compounds **11** and **12**.

Compounds **6**, **10** and **11** caused 100% mortality after 12 h against *A. monuste orseis*. After 24 h, compounds **8**, **9** and **12** also presented similar activity (92.50% and 95.00%) to the commercial pyrethroid. Compounds **6**, **8**, **9 **and **11** presented high toxicity 12 h after application against *P. Americana *(mean 95.42% 24 h after application), pointing to a rapid mechanism of action similar to that of permethrin. Only compounds **7a **and **7b** presented an increase in toxicity from 12 h (79.33% and 67.00%) to 24 h (96.00% and 71.00%) respectively. 

Compounds **6** and **11** were the most active against the insect species evaluated and presented almost complete mortality even after 12 hours of application. We observed that all the substances, except **8**, **9** and **12**, showed fast insecticidal action, as the highest amount of insects deaths occurred 12 h after application. Substances **8**, **9** and **12** presented the highest activity against *A. monuste* only after 24 h.

**Table 2:** Contact toxicity of the synthetic compounds at concentration of 5 μg (*P. americana*), 10 µg (*A. monuste*) and 50 μg (*A. obtectus* and *S. zeamais*) of compound per mg of insect.

Treatment (compound)	Mean percent mortality *										
	*A. obtectus*			*S. zeamais*			*A. monuste orseis*			*P. americana*	
	12 h	24 h		12 h	24 h		12 h	24 h		12 h	24 h
Permethrin ^#^	100.0 aA	100.0 aA		85.5 bA	90.0 aA		100.0 aA	100.0 aA		87.5 bA	97.5 aA
**6**	100.0 aA	100.0 aA		85.00 aA	90.00 aA		100.00 aA	100.00 aA		100.00 aA	100.00 aA
**7a**	100.0 aA	100.0 aA		45.00 bB	47.50 bB		27.50 cD	47.50 bC		79.33 aABC	96.00 aAB
**7b**	90.00 aA	97.50 aA		42.50 cB	45.00 cB		50.00 cC	67.50 bB		67.00 bC	71.00 aCD
**8**	87.50 aA	95.00 aA		95.00 aA	95.00 aA		62.50 bBC	95.00 aA		100.00 aA	100.00 aA
**9**	82.50 aA	92.50 aA		92.50 aA	97.50 aA		75.00 aB	95.00 aA		92.00 aAB	92.00 aABC
**10**	97.50 aA	97.50 aA		82.50 aA	90.00 aA		100.00 aA	100.00 aA		67.00 bC	67.00 bD
**11**	100.0 aA	100.0 aA		97.50 aA	100.00 aA		100.00 aA	100.00 aA		100.00 aA	100.00 aA
**12**	97.50 aA	97.50 aA		97.50 aA	100.00 aA		75.00 aB	92.50 aA		75.00 aBC	75.00 aBCD
Control ^§^	0.00 aB	3.33 aB		0.00 aC	3.33 aC		11.67 aD	13.33 aD		4.00 aD	6.00 aE

* Means followed by the same lower-case letter in a row or by the same upper-case letter in a column are not significantly different by the Tukey test at *p* > 0.05. ^§^ Only ketone was used in the control. ^#^ Commercial insecticide.

Therefore, the substances **6**, **8**, **9** and **11** have a potential use in controlling pests of stored products (*A. obtectus* and *Sitophilus zeamais*), caterpillars (*A. monuste*) and cockroaches (*P. americana*). These results are promising since the discovery of new substances with insecticidal action is an alternative to be used in pest management to avoid selection of resistant populations to insecticides as with *P. americana * [28] and *S. zeamais* [29]. 

Moreover, as these substances present fast insecticide action they present potential use in pest control when infestations are high. Herbivores such as *A. obtectus*, *A. monuste*, and *S. zeamais* could be controlled before they cause damage to plants, or household pests (cockroaches) before they transmit diseases to humans [30,31].

## 3. Experimental

### 3.1. General Procedures

DCM and THF were dried as described in the literature [32]. The melting points were determined on an Electrothermal digital (MQAPF-301) apparatus without correction. Infrared spectra were recorded on a Perkin Elmer Paragon 1000 FTIR grating spectrophotometer using potassium bromide disks and scanning from 4000 to 625 cm^−1^. ^1^H- and ^13^C-NMR spectra were recorded in a Varian Mercury (300 MHz) spectrometer. Tetramethylsilane (SiMe_4_) was used as internal standard (*δ *= 0). GC-MS was conducted with a Shimadzu QP5050A spectrometer, using a glass capillary column (25 m × 0.25 mm) DB-1. The elementary analysis was carried out in a Perkin Elmer 2400 instument. The reactions were monitored by thin layer chromatography (TLC) using plates coated with 60GF_254_ silica-gel (POLYGRAM-UV_254_ 0.25 mm MACHEREY—NAGEL).

### 3.2. Synthetic Procedures

*4-(1'-Hydroxy-1'-methylethyl)tetrahydrofuran-2-one* (**3**). A solution of furan-2(5*H*)-one (4.5 g; 53.52 mmol) in isopropyl alcohol (100 mL) was degassed in a quartz tube by a steady flow of nitrogen for 1 h. The tube was stoppered and irradiated for 12 h under four low pressure mercury lamps (4 × 15 W). The solvent was removed under reduced pressure and the residue purified by column chromatography (hexane-ethyl acetate, 1:2 v/v) to afford **3** (7.7 g; 53.52 mmol) in 100% yield as a yellowish oil. TLC: R_f_ = 0.52 (hexane-ethyl acetate, 1:2 v/v); IR (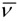, cm^−1^): 3426, 2978, 2928, 1774, 1378, 1186, 1016, 956, 850. ^1^H-NMR (CDCl_3_) *δ* (m, l, *J *(Hz), atrib.): 1.28 (m, 6H, 2 × CH_3_), 2.61 (m, 3H, H3, H3' and H4), 2.8 (s, 1H, OH), 4.4 (m, 2H, H5 and H5'); ^13^C-NMR (CDCl_3_): *δ* 27.9 (CH_3_), 28.3 (CH_3_), 30.1 (C3), 46.1 (C4), 69.7 (C5), 70.0 (C-OH), 178.0 (C=O); MS, *m/z* (100%): [*M_•_*^+^] 144 (1.43), 126 (3.40), 111 (3.60), 85 (56.39), 59 (100.00).

*4-Isopropenyltetrahydrofuran-2-one* (**4a**) and *4-(1-methylethylidene)tetrahydrofuran-2-one* (**4b**). A suspension of PCl_5_ (20 g; 95.92 mmol) in anhydrous DCM (25 mL) was added to an ice cooled solution of **3** (7 g; 48.61 mmol) in anhydrous DCM (40 mL). The reaction mixture was stirred for 5 min, quenched with distilled water (150 mL), the organic phase separated, and the aqueous layer extracted with DCM (3 × 50 mL). The combined organic phases was washed with brine (3 × 25 mL), dried with anhydrous magnesium sulfate, filtered, and concentrated under reduced pressure. Flash column chromatography (hexane:ethyl acetate, 1:1 v/v) of the residue gave a mixture of the isomers (**4a**) and (**4b**) in 90% yield (5.5 g). Compound **4a**: TLC: R_f_ = 0.65 (hexane-ethyl acetate, 1:1 v/v); IR (KBr, 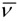, cm^−1^): 3083, 2975, 2916, 1794, 1650, 1179, 1021, 900; ^1^H-NMR (CDCl_3_) *δ* (m, l, *J*(Hz), atrib.): 1.73 (s, 3H, CH_3_), 2.43 (dd, 1H, *J*_gem_ = 17.1, *J*_3–4_ = 8.5, H3), 2.62 (dd, 1H, *J*_gem_ = 17.1 and *J*_3'-4_ = 8.5, H3), 3.15 (m, 1H, H4), 4.07 (dd, 1H, *J*_gem_ = 8.8 and *J*_5–4_ = 7.8, H5), 4.42 (dd, 1H, *J*_gem_ = 8.8 and J_5'-4_ = 7.8, H5'), 4.85 (m, 2H, =CH_2_); ^13^C-NMR (CDCl_3_): *δ* 20.5 (CH_3_), 33.2 (C3), 42.5 (C4), 71.9 (C5), 112.4 (=CH_2_), 176.9 (C=O); MS, *m/z* (100%): [*M_•_*^+^] 126 (12.36), 125 (28.86), 95 (22.98), 68 (88.19), 67 (100.00), 59 (34.65). Compound **4b**: TLC: R_f_ = 0.68 (hexane-ethyl acetate, 1:1 v/v); IR (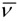, cm^−1^): 2974, 2912, 1784, 1650, 1362, 1180, 1025, 847, 558; ^1^H-NMR (CDCl_3_) *δ* (m, l, *J*(Hz), atrib.): 1.60 (m, 3H, CH_3_), 1.68 (m, 3H, CH_3_'), 3.13 (m, 2H, H3 e H3'), 4.83 (m, 2H, H5 and H5'); ^13^C-NMR (CDCl_3_): *δ* 19.6 (CH_3_), 21.6 (CH_3_'), 32.4 (C3), 71.6 (C5), 121.8 (C4), 126.8 (C6), 176.7 (C=O).

*4-(1'-Bromo-1'-methylethyl)tetrahydrofuran-2-one* (**5**) from the mixture of **4a **and **4b**. A solution of PBr_3_ (1.6 mL, 17.02 mmol) in DCM (10 mL) was added dropwise to a suspension of SiO_2_ (15 g) in DCM (50 mL) containing the isomers **4a** and **4b** (5.5 g; 43.65 mmol). The mixture was stirred for 1 h, filtered under vacuum and the SiO_2_ washed with DCM (30 mL). The filtrate was washed with NaHCO_3_ 10% (2 × 20 mL) and brine (2 × 20 mL). The organic phase was dried with MgSO_4_, filtered, concentrated under reduced pressure and the residue purified by flash column chromatography (hexane:ethyl acetate, 1:1 v/v) to afford **5** (3.5 g; 17.02 mmol) in 39% yield as a white solid. TLC: R_f_ = 0.52 (hexane-ethyl acetate, 1:1 v/v); IR (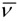, cm^−1^): 2973, 2917, 1779, 1373, 1175, 1115, 1027, 629; ^1^H-NMR (CDCl_3_) *δ* (m, l, *J*(Hz), atrib.): 1.75 (m, 6H, 2 × CH_3_), 2.64 (m, 3H, H3, H3' and H4), 4.29 (m, 1H, H5), 4.45 (m, 1H, H5'); ^13^C-NMR (CDCl_3_): *δ* 31.9 (C3), 32.1 (CH_3_), 32.4 (CH_3_), 48.3 (C4), 66.6 (C-Br), 70.6 (C5), 175.8 (C=O); MS, *m/z* (100%): [*M_•_*^+^+ 2] 209 (1.63), [*M_•_*^+^] 207 (1.67), 126 (17.59), 111 (60.31), 83 (100.00), 68 (54.27).

*4-(1'-Bromo-1'-methylethyl)tetrahydrofuran-2-one *(**5**) from *4-(1′-hydroxy-1′-methylethyl)tetrahydrofuran-2-one* (**3**). A solution of PBr_3_ (2.0 mL; 21.28 mmol) in DCM (10 mL) was added dropwise to a solution of **3** (7.00 g; 48.61 mmol), and pyridine (4.0 mmL; 49.56 mmol) in DCM (25 mL). The reaction mixture was stirred for 1 h, and a further dropwise addition of PBr_3_ (2.0 mL; 21.28) in DCM (10 mL) was performed. After addition of SiO_2_ (15 g), the mixture was stirred for a further 40 min. The reaction mixture was filtered under vacuum and the silica was washed with DCM (30 mL). The filtrate was washed with NaHCO_3_ 10% (2 × 20 mL) and brine (2 × 20 mL). The organic phase was dried with MgSO_4_, filtered, concentrated under reduced pressure and the residue purified by flash column chromatography (hexane:ethyl acetate, 1:1 v/v) to afford **5** (5.03 g; 24.3 mmol) in 50% yield as a white solid. TLC: Rf = 0.52 (hexane-ethyl acetate, 1:1 v/v); IR (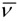, cm^−1^): 2973, 2917, 1779, 1373, 1175, 1115, 1027, 629; ^1^H-NMR (CDCl_3_) *δ* (m, l, *J*(Hz), atrib.): 1.75 (m, 6H, 2 × CH_3_), 2.64 (m, 3H, H3, H3' e H4), 4.29 (m, 1H, H5), 4.45 (m, 1H, H5'); ^13^C-NMR (CDCl_3_): *δ* 31.8 (C3), 32.0 (CH3), 32.3 (CH3), 48.2 (C4), 66.5 (C-Br), 70.5 (C5), 175.7 (C=O); MS, *m/z* (100%): [*M_•_*^+^+ 2] 209 (1.63), [*M_•_*^+^] 207 (1.67), 126 (17.59), 111 (60.31), 83(100.00), 68 (54.27).

*6,6-Dimethyl-3-oxabicyclo[3.1.0]hexan-2-one* (**6**). A solution of **5** (2.00 g; 9.66 mmol) in anhydrous THF (20 mL) was added dropwise to an ice cooled suspension of potassium *tert*-butoxide (2.20 g; 19.64 mmol) in anhydrous THF (30 mL). The reaction mixture was stirred for 5 min, quenched with NH_4_Cl (30 mL) and extracted with DCM (3 × 50 mL). The combined organic phases was dried with anhydrous MgSO_4_, filtered and concentrated under reduced pressure. The residue was purified by flash column chromatography (hexane:ethyl acetate, 1:1 v/v) to afford **6** (0.4870 g; 3.87 mmol) in 40% yield as a colorless oil. TLC: Rf = 0.52 (hexane-ethyl acetate , 1:1 v/v); IR (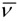, cm^−1^): 3068, 2961, 2909, 1774, 1368, 1186, 1046, 975, 892; ^1^H-NMR (CDCl_3_) *δ* (m, l, *J*(Hz), atrib.): 1.10 (s, 6H, 2 × CH_3_), 1.88 (dd, 1H, *J*_3-4 _= 6.3 e *J*_3–5’ _= 0.9, H3), 2.01 (ddd, 1H, *J*_3–4_ = 6.3, *J*_4-5_ = 5.2 e *J*_4–5'_ = 1.2, H4), 4.10 (dt, 1H, *J*_gem_ = 9.9, *J*_3–5'_ = 0.9 e *J*_4–5'_ = 1.2, H5’), 4.31 (dd, 1H, *J*_gem_ = 9.9 and *J*_4–5_ = 5.2, H5); ^13^C-NMR (CDCl_3_): *δ* 14.6 (CH_3_), 23.3 [C(CH_3_)_2_], 25.4 (CH_3_), 30.3 (C4), 30.7 (C3), 66.8 (C5), 175.3 (C=O). MS, *m/z *(100%): [*M_•_*^+^] 126 (3.67), 125 (9.74), 111 (18.33), 97 (25.60), 85 (28.50), 83 (31.98), 71 (53.30), 69 (47.64), 57 (100.00), 55 (67.45).

### 3.3. General Method to Preparation of Alkenes ***8*** to ***12***

To a suspension of the Wittig salt (3.53 mmol) in dry THF (5 mL) under nitrogen at 0 °C *n*-butyl lithium was added (2.0 M in hexane, 1,8 mL ; 3.60 mmol). The reaction mixture was stirred for 20 min, and the aldehyde (**7**) (0.50 g; 3.20 mmol) in dry THF (5 mL) was added. After 30 min at 0 °C, the cooling bath was removed and the mixture was stirred for 3 h. The reaction was quenched with saturated aqueous ammonium chloride (10 mL), the THF was evaporated under reduced pressure and the aqueous residue was extracted with diethyl ether (5 × 10 mL). The combined organic layers was dried with anhydrous magnesium sulfate, filtered and concentrated in the rotary evaporator to give a yellow oil. The oil was purified by flash column chromatography (hexane:ethyl ether, 9:1 v/v). This procedure was used for the preparation of the compounds **8** to **12**. The quantity of Wittig salts and yield for each reaction are shown in Table 1.

*Mixture of isomers (Z)-(***8a***) and (E)-(***8b***) of methyl (1S,3S)-3-[2-(2-methoxyphenyl)ethen-1-yl]-2,2-dimethylcyclopropane-1-carboxylate*. TLC: Rf = 0.45 (hexane-diethyl ether, 9:1 v/v); Specific rotation: [*α*]*_D_*^20^= −84.4° (c = 1.60, acetone); IR (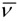, cm^−1^): 2950, 1724, 1598, 1438, 1244, 1167, 1029, 752; ^1^H-NMR (CDCl_3_) *δ* (m, l, *J*(Hz), atrib.): 1.20 (s, 6H, 2 × CH_3_), 1.25 (s, 3H, CH_3_), 1.30 (s, 3H, CH_3_), 1.57 (d, 1H, *J*_1-3*cis*_ = 5.4, H1*_cis_*), 1.69 (d, 1H, *J*_1-3*trans*_ = 5.4, H1*_trans_*), 2.26 (dd, 1H, *J*_1-3*trans*_ = 5.4 e *J*_3-4*trans*_ = 8.7, H3*_trans_*), 2.34 (dd, 1H, *J*_1-3* cis*_ = 5.4 e *J*_3-4* cis*_ = 9.0, H3*_cis_*), 3.65 (s, 3H, OCH_3_), 3.70 (s, 3H, OCH_3_), 3.83 (s, 3H, CH_3_O-Ar), 3.83 (s, 6H, CH_3_O-Ar), 5.45 (dd, 1H, *J*_3-4*cis*_ = 9.0 e *J*_4-5*cis*_ = 11.5, H4*_cis_*), 5.96 (dd, 1H, *J*_3-4*trans*_ = 8.7 e *J*_4-5*trans*_ = 15.9, H4*_trans_*), 6.68 (d, 1H, *J*_4-5*cis*_ = 11.5, H5*_cis_*), 6.92 (m, 5H, H5*_trans_*, H3' e H5'), 7.30 (m, 4H, H4' e H6'); ^13^C-NMR (CDCl_3_): *δ* 20.4 (CH_3_), 20.7 (CH_3_), 22.4 (CH_3_), 22.5 (CH_3_), 29.7 [C(CH_3_)_2_], 28.8 [C(CH_3_)_2_], 33.7 (C3*_cis_*), 34.6 (C1*_trans_*), 35.7 (C1*_cis_*), 37.6 (C3*_trans_*), 51.7 (OCH_3_), 51.8 (OCH_3_), 55.7 (CH_3_OAr), 55.8 (CH_3_OAr), 110.5–130.2 (C4, C5 e Ar), 156.5 (C2'), 157.1 (C2'), 172.5 (C=O), 172.7 (C=O); MS, *m/z* (100%): [*M_•_*^+^] 260 (35.02), 201 (100.00), 185 (46.55), 121 (56.32), 91 (95.16), 77 (57.62), 41 (73.00).

*Mixture of isomers (Z)-(***9a***) and (E)-(***9b***) of methyl (1S,3S)-3-[2-(3-methoxyphenyl)ethen-1-yl]-2,2-dimethylcyclopropane-1-carboxylate*. TLC: Rf = 0.45 (hexane-diethyl ether, 9:1 v/v); Specific rotation: [*α*]*_D_*^20^= −80.9° (c = 1.10, acetone); IR (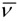, cm^−1^): 2948, 1728, 1438, 1219, 767, 751; ^1^H-NMR (CDCl_3_) *δ* (m, l, *J *(Hz), atrib.): 1.24 (s, 6H, CH_3 *cis*_), 1.26 (s, 3H, CH_3 *trans*_), 1.28 (s, 6H, CH_3 *cis*_), 1.34 (s, 3H, CH_3 *trans*_), 1.60 (d, 2H, *J*_1-3*cis*_ = 5.7, H1*_cis_*), 1.73 (d, 1H, *J*_1-3*trans*_ = 5.7, H1*_trans_*), 2.27 (m, 3H, H3*_cis_* e H3*_trans_*), 3.67 (s, 6H, OCH_3* cis*_), 3.71 (s, 3H, OCH_3 *trans*_), 5.55 (dd, 2H, *J*_3-4*cis*_ = 9.0 e *J*_4-5*cis*_ = 11.4, H4*_cis_*), 5.97 (dd, 1H, *J*_3-4*trans*_ = 9.0 e *J*_4-5*trans*_= 15.9, H4*_trans_*), 6.67 (d, 2H, *J*_4-5*cis*_ = 11,4, H5*_cis_*), 6.95 (d, 1H, *J*_4-5*trans*_ = 15.9, H5*_trans_*), 7.25 (m, 12H, Ar*_cis_* e Ar*_trans_*)*; ^13^C-NMR (CDCl_3_): δ 20.4 (2 × CH_3_), 20.6 (CH_3_), 22.4 (CH_3_), 29.7 [2 × C(CH3)2], 33.2 (C3*_cis_*), 34.8 (C1*_trans_*), 35.7 (C1*_cis_*), 37.0 (C3*_trans_*), 51.8 (2 × OCH_3_), 126.5–135.3 (C4, C5 e Ar), 172.4 (2 × C=O); MS, *m/z* (100%): [*M_•_*^+^] 260 (26.10), 201 (61.42), 185 (58.24), 159 (60.84), 115 (77.52), 102 (100.00), 91 (67.98), 77 (68.16), 41 (72.87). 

*Mixture of isomers (Z)-(***10a***) and (E)-(***10b***) of methyl (1S,3S)-3-[2-(2-chlorophenyl)ethen-1-yl]-2,2-dimethylcyclopropane-1-carboxylate*. TLC: Rf = 0.55 (hexane-diethyl ether, 9:1 v/v); Specific rotation: [*α*]*_D_*^20^= −80.6° (c= 1.32, acetone); IR (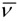, cm^−1^): 2956, 2752, 1738, 1714, 1434, 1237, 1173, 1112, 975; ^1^H-NMR (CDCl_3_) *δ* (m, l, *J*(Hz), atrib.): 1.20 (s, 6H, 2 × CH_3_), 1.25 (s, 6H, 2 × CH_3_) 1.57 (d, 1H, *J*_1-3*cis*_ = 5.4, H1*_cis_*), 1.67 (d, 1H, *J*_1-3*trans*_ = 5.7, H1*_trans_*), 2.22 (dd, 1H, *J*_1-3*trans*_ = 5.7 e *J*_3-4*trans*_ = 8.4, H3*_trans_*), 2.45 (dd, 1H, *J*_1-3*cis*_ = 5.4 e *J*_3-4*cis*_ = 8.7, H3*_cis_*), 3.67 (s, 3H, OCH_3_), 3.69 (s, 3H, OCH_3_), 3.80 (s, 6H, 2 × CH_3_O-Ar), 5.40 (dd, 1H, *J*_3-4*cis*_ = 8.7 e *J*_4-5*cis*_ = 11.7, H4*_cis_*), 5.95 (dd, 1H, *J*_3-4*trans*_ = 8.4 e *J*_4-5*trans*_ = 15.9, H4*_trans_*), 6.54 (m, 2H, H5*_trans _*e H5*_cis_*), 6.90 (m, 6H, H2', H4' e H6'), 7.23 (m, 2H, H5’); ^13^C-NMR (CDCl_3_): *δ* 20.8 (CH_3_), 21.1 (CH_3_), 33.4 (C2), 34.3 (C1), 42.2 (C3), 52.2 (OCH_3_), 170.4 (COOCH_3_), 199.0 (HC=O); MS, *m/z* (100%): [*M_•_*^+^+2] 266 (6.29), [*M_•_*^+^] 264 (19.09), 207 (28.14), 205 (100.00), 139 (43.93), 125 (84.02), 115 (31.50), 77 (25.86).

*Mixture of isomers (Z)-(***11a***) and (E)-(***11b***) of methyl (1S,3S)-3-[2-(pentafluorophenyl)ethen-1-yl]-2,2-dimethylcyclopropane-1-carboxylate*. TLC: Rf = 0.63 (hexane-diethyl ether, 9:1 v/v); Specific rotation: [*α*]*_D_*^20^= −74.6° (c = 2.60, acetone); IR (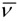, cm^−1^): 2952, 1734, 1651, 1521, 1495, 1219, 1171, 1002, 869; ^1^H-NMR (CDCl_3_) *δ* (m, l, *J*(Hz), atrib.): 1.23 (s, 6H, 2 × CH_3_), 1.34 (s, 6H, 2 × CH_3_) 1.65 (d, 1H, *J*_1-3*cis*_ = 5.4, H1*_cis_*), 1.75 (d, 1H, *J*_1-3*trans*_ = 5.4, H1*_trans_*), 2.16 (m, 1H, H3*_cis_*), 2.23 (m, 1H, H3*_trans_*), 3.65 (s, 3H, OCH_3_), 3.67 (s, 3H, OCH_3_), 5.75 (dd, 1H, *J*_3-4*cis*_ = 9.6 e *J*_4-5*cis*_ = 11.1, H4*_cis_*), 6.14 (d, 1H, *J*_4-5*cis*_ = 11.1, H5*_cis_*), 6.27 (dd, 1H, *J*_3-4*trans*_ = 9.0 e *J*_4-5*trans*_ = 16.2, H4*_trans_*), 6.46 (d, 1H, *J*_4-5*trans*_ = 16.2, H5*_trans_*); ^13^C-NMR (CDCl_3_): δ 20.5 (2 × CH_3_), 22.3 (CH_3_), 22.5 (CH_3_), 29.8 [C(CH_3_)_2_], 30.0 [C(CH_3_)_2_], 34.1 (C3*_cis_*), 34.2 (C1*_trans_*), 35.7 (C1*_cis_*), 37.7 (C3*_trans_*), 51.9 (OCH_3_), 52.0 (OCH_3_), 114.7 (C5*_cis_*), 115.8 (C5*_trans_*), 137.4 (C4*_cis_* e C4*_trans_*), 172.0 (2 × C=O); MS, *m/z* (100%): [*M_•_*^+^] 320 (8.37), 261 (100.00), 195 (24.67), 181 (98.29), 139 (38.03), 59 (38.77), 41 (57.36).

*Mixture of isomers (Z)-(***12a***) and (E)-(***12b***) of methyl (1S,3S)-3-[2-(4-ethoxyphenyl)ethen-1-yl]-2,2-dimethylcyclopropane-1-carboxylate*. TLC: Rf = 0.52 (hexane-diethyl ether, 9:1 v/v); Specific rotation: [*α*]*_D_*^20^= −71.4° (c= 2.80, acetone); IR (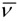, cm^−1^): 2979, 2949, 1725, 1607, 1510, 1245, 1167, 1048, 841, 732; ^1^H-NMR (CDCl_3_) *δ* (m, l, *J*(Hz), atrib.): 1.20 (s, 3H, CH_3_), 1.21 (s, 3H, CH_3_), 1.30 (s, 3H, CH_3_), 1.32 (s, 3H, CH_3_), 1.54 (d, 1H, *J*_1-3*cis*_ = 5.4, H1*_cis_*), 1.64 (d, 1H, *J*_1-3*trans*_ = 5.4, H1*_trans_*), 2.18 (m, 1H, H3*_trans_*), 2.40 (m, 1H, H3*_cis_*), 3.67 (s, 3H, OCH_3_), 3.69 (s, 3H, OCH_3_), 4.03 (m, 4H, 2 × OCH_2_), 5.30 (dd, 1H, *J*_3-4*cis*_ = 8.4 e *J*_4-5*cis*_ = 11.5, H4*_cis_*), 5.82 (dd, 1H, *J*_3-4*trans*_ = 8.7 e *J*_4-5*trans*_ = 15.6, H4*_trans_*), 6.46 (m, 2H, H5*_trans _*e H5*_cis_*), 6.85 ( m, 4H, H3'), 7.25 (m, 4H, H2'); ^13^C-NMR (CDCl_3_): δ 15.1 (2 × OCH_2_CH_3_), 20.4 (2 × CH_3_), 22.4 (2 × CH_3_), 29.5 [C(CH_3_)_2_], 39.8 [C(CH_3_)_2_], 33.4 (C3*_cis_*), 34.5 (C1_t_*_rans_*), 35.7 (C1*_cis_*), 37.1 (C3*_trans_*), 51.8 (2 × OCH_3_), 63.36 (2 × OCH_2_), 114.4 (C3'), 114.7 (C3'), 125.0 (C4*_trans_*), 126.7 (C4*_cis_*), 127.2 (2 × C1'), 129.8 (C2'), 130.2 (C2'), 131.5 (C5), 131.6 (C5), 158.1 (C4'), 158.5 (C4'), 172.7 (2 × C=O); MS, *m/z* (100%): [*M_•_*^+^] 274 (51.44), 215 (100.00), 199 (35.28), 107 (50.20), 77 (44.56), 29 (62.83).

### 3.4. Bioassays

To evaluate the insecticidal activity of the synthesized compounds, biological assays were conducted with *A. obtectus*, adults of *S. zeamais*, second-instar larvae of *A. monuste orseis*, and second-instar nymph of *P. americana*.

The experimental design was completely randomized with four replicates. Each experimental unit consisted of a glass Petri dish (9.5 cm × 2.0 cm) containing ten insects. The average weight of each insect species was obtained by measuring, on an analytical balance, the mass of ten groups containing 10 insects each. Bioassays were conducted by topical application. For each individual insect was applied on the thoracic tergite, via a 10 µL Hamilton micro syringe, 0.5 µL of a solution of the test compound, dissolved in acetone, corresponding to dose of 5 (for *P. americana*), 10 (for *A. monuste orseis*) and 50 µg of compound per mg of the insect (for *A. obtectus *e *S. zeamais*). In a control experiment, carried out under the same conditions, 0.5 µL of acetone was applied on each insect. After application, the insects were kept in individual Petri dishes and *A. monuste orseis* and *P.americana* were supplied with appropriate food. No food was supplied to *A. obtectus *e *S. zeamais*.

In all cases the Petri dishes were placed in an incubator at 25 ± 0.5 °C, 75 ± 5% relative humidity, with a photoperiod of 12 h. The mortality counts were made after 12 h after treatment. Mortality included dead individuals and those without movements. Mortality data were analyzed using Tukey test at 0.05 probability level.

## 4. Conclusions

In conclusion, we have successfully synthezed 6,6-dimethyl-3-oxabicyclo[3.1.0]hexan-2-one (**6**) from the readily available furfural and of five novel pyrethroids **8**–**12** from D-mannitol. These compounds were evaluated for their biological activity against some key pests common in Brazil: *A. obtectus*, *S. zeamais*, *A. monuste orseis*, and *P. americana*. The results indicate that all compounds show insecticidal activity, and compounds **6 ** and **11** presented insecticidal activity statistically not different from the commercial insecticide for all species tested.
